# Energy band alignment at the heterointerface between CdS and Ag-alloyed CZTS

**DOI:** 10.1038/s41598-020-73828-0

**Published:** 2020-10-27

**Authors:** Mungunshagai Gansukh, Zheshen Li, Moises Espindola Rodriguez, Sara Engberg, Filipe Mesquita Alves Martinho, Simon López Mariño, Eugen Stamate, Jørgen Schou, Ole Hansen, Stela Canulescu

**Affiliations:** 1 Department of Photonics Engineering, Technical University of Denmark, 4000 Roskilde, Denmark; 2 ISA, Department of Physics and Astronomy, Aarhus University, 8000 Aarhus C, Denmark; 3 Department of Energy Conversion and Storage, Technical University of Denmark, 4000 Roskilde, Denmark; 4 DTU Nanolab, Technical University of Denmark, 2800 Kgs. Lyngby, Denmark

**Keywords:** Solar cells, Laser-produced plasmas, Surface spectroscopy, Electronic properties and materials

## Abstract

Energy band alignment at the heterointerface between CdS and kesterite Cu_2_ZnSnS_4_ (CZTS) and its alloys plays a crucial role in determining the efficiency of the solar cells. Whereas Ag alloying of CZTS has been shown to reduce anti-site defects in the bulk and thus rise the efficiency, the electronic properties at the interface with the CdS buffer layer have not been extensively investigated. In this work, we present a detailed study on the band alignment between n-CdS and p-CZTS upon Ag alloying by depth-profiling ultraviolet photoelectron spectroscopy (UPS) and X-ray photoelectron spectroscopy (XPS). Our findings indicate that core-level peaks and the valence band edge of CdS exhibit a significant shift to a lower energy (larger than 0.4 eV) upon the etching of the CdS layer, which can be assigned due to band bending and chemical shift induced by a change in the chemical composition across the interface. Using a simplified model based on charge depletion layer conservation, a significantly larger total charge region depletion width was determined in Ag-alloyed CZTS as compared to its undoped counterpart. Our findings reveal a cliff-like band alignment at both CdS/CZTS and CdS/Ag-CZTS heterointerfaces. However, the conduction-band offset decreases by more than 0.1 eV upon Ag alloying of CZTS. The approach demonstrated here enables nanometer-scale depth profiling of the electronic structure of the p–n junction and can be universally applied to study entirely new platforms of oxide/chalcogenide heterostructures for next-generation optoelectronic devices.

## Introduction

The Cu_2_ZnSn(S_x_Se_1−x_)_4_ (CZTSSe) solar cells have currently reached a power conversion efficiency of 12.6%^1^, which is slightly more than half the efficiency of CuIn_x_Ga_1−x_Se_2_ (CIGS) solar cells^2^. The progressive improvement in the CZTS efficiency has been limited by difficulties related to the low open-circuit voltage (*V*_oc_). The *V*_oc_ deficit of the CZTS devices (defined as *E*_g_/*q*-*V*_oc_, where *E*_g_ is the optical bandgap and *q* is the elementary charge) is substantially higher than that of CdTe and CIGS solar cells with absorbers of comparable band gap^3^. Alloying of the absorber has been proposed as an approach to address the issue of the low *V*_oc_, typically associated with a high density of defects in the bulk, which results in a reduced minority carrier lifetime^4–6^. In particular, alloying of CZTS with Ag has been theoretically predicted to significantly reduce the density of the Cu_Zn_ anti-site defects^5^. In pure CZTS, Cu and Zn atoms are nearly completely site-disordered upon high temperature processing. In the case of Ag_2_ZnSnS_4_ (AZTS), the swap between Ag with Zn is no longer favored because of the large radius mismatch (Ag has a covalent radius that is ~ 15% larger than that Cu)^5^. The high formation energy of the Ag_Zn_ anti-site defects in AZTS results in a reduced defect density and enhanced *V*_oc_^7^. Nevertheless, there is an optimum value for Ag alloying of CZTS because: i) a high Ag content reduces the p-type conductivity and leads to an n-type conductivity in the case of AZTS, and ii) the band gap increases from 1.5 eV for pure CZTS to 2.1 eV for AZTS, which is no longer an optimum value for a single junction solar cell. Therefore, optimized solar cells of (Ag_x_Cu_1−x_)_2_ZnSnS_4_ (ACZTS) can benefit from a large *V*_oc_ while still maintaining a near optimum band gap. For example, an increase in the device performance from 4.9 to 7.2% has been reported for ACZTS solar cells^8^. Although experimental studies have shown the benefits of Ag alloying of CZTS, an experimental determination of the band alignment at the heterointerface with CdS upon Ag alloying of sulfides is still lacking. The band alignment at the heterointerface is crucial for device performance, and to achieve high-performance solar cells the focus should be directed towards interface engineering. In particular, for a high-efficiency device, the conduction band minimum (CBM) of the n-type semiconductor must be slightly higher than that of the p-type semiconductor at the interface, i.e., a *spike-like* or positive conduction band alignment, or at least flat-band. For instance, a positive conduction band offset (CBO) of ~ 0.25 eV has been experimentally estimated for the CdS/CIGS device with a power conversion efficiency better than 18%^9^. CZTS devices with efficiencies exceeding 7% show a flat-band CBO^10,11^, while most cases where the efficiency exceeds 10%, such as CZTSe, a spike-like CBO was estimated^12^. The spike-like barrier acts as a notch against photo-generated carriers in the absorber layer preventing recombination losses^13^. Spike barriers up to 0.4 eV are considered beneficial for solar cells, while larger CBO values result in a decrease of the short-circuit current (J_sc_) and efficiency^13,14^. On the other hand, when the CBO is negative, that is, the position of the CBM of the n-type semiconductor is lower than that of the p-type semiconductor (*cliff-lik*e band alignment), the cell performance is poor due to enhanced carrier recombination at trap states near the interface^5,13^. Consequently, *V*_oc_ decreases with increasing the absolute value of the CBO^13^. The latter case applies for poorly performing CZTS devices^15^.

In this paper, we perform a systematic study on the band alignment at the CdS/CZTS and CdS/ACZTS interfaces using X-ray photoemission spectroscopy (XPS) and ultraviolet photoemission spectroscopy (UPS). Moreover, the conduction band states of CdS, CZTS and ACZTS were studied by S L_2,3_ X-ray absorption spectroscopy (XAS). Our approach in measuring the band offset was to perform a mild Ar^+^ ion etching of the CdS overlayer, as illustrated in Fig. 6 (a). Photoemission spectra were then obtained after each etching step to observe the development of the electronic structure at the heterojunction and to obtain a direct determination of the valence band offset (VBO). Simultaneously, the binding energy of the core levels obtained by XPS was used to estimate the band bending at the heterointerface. Finally, based on a combination of the different techniques, the band alignment at the CdS/CZTS and CdS/ACZTS heterojunction interfaces is revealed.

## Experimental section

### Materials

CZTS and ACZTS films were grown by pulsed laser deposition (PLD), using a similar procedure which led to an efficiency of solar cells produced by PLD of 5.4%^16^. The CZTS and ACZTS films were deposited at room temperature on Mo-coated soda-lime glass substrates placed at a distance of 5 cm from the target. The precursors were made by ablation of CZTS and ACZTS targets in vacuum using a KrF excimer laser (λ = 248 nm, τ = 20 ns, ν = 15 Hz) and a laser fluence of ~ 0.6 J/cm^2^. The CZTS and ACZTS stoichiometric targets (from American Elements) consist of a mixture of their corresponding binary sulfide phases. The targets were rotated during deposition and the laser beam was rastered across the target to ensure uniform ablation. The ACZTS had a metal ratio of Ag/(Ag + Cu) ~ 0.5. After the growth, the films were thermally annealed in a graphite box in a furnace at a temperature of 575 °C in the presence of S and SnS powders. The absorbers were submitted to a (NH_4_)_2_S etching step for a duration of 5 min^17^, and immediately after coated with a thin layer of CdS by chemical bath deposition^18^. The CdS layer was deposited simultaneously by placing the CZTS and ACZTS samples in the same bath. The samples were then vacuum-sealed and transported to the beamline for measurements.

### Methods

The UPS, XPS and XAS measurements were carried out at the Matline end-station at the ASTRID storage ring facility, ISA, Denmark. The measurements were taken in a UHV chamber (base pressure below 1 × 10^–9^ Torr) equipped with a Scienta electron energy analyzer. The UPS spectra were obtained using excitation energies of 60 and 130 eV. The XPS core level spectra were recorded as following: 60 and 130 eV excitation energy for Cd 4d and Zn 3d, 400 eV excitation energy for Ag 3d, and 220 eV excitation energy for S2p. Since the Cd 4d and Zn 3d XPS peaks are located close to the valence band, they were conveniently chosen to monitor XPS and valence band spectra in one scan, rather than measuring the Cd 3d and Zn 2p peaks used in the conventional XPS. In addition, when the Cd 4d and Zn 3d peaks were recorded in one spectra, any possible charging effects at a given depth are negligible. The beam spot size was ~ 0.7 × 1 mm^2^. To access the interface, a mild sputtering of the CdS layer was carried out using an Ar^+^ ion gun (from Varian) at an angle of 40 degrees with respect to sample surface and ion energy varying from 0.5 to 1 keV for a duration of 20 min. To enable a direct comparison between samples, the etching of the CdS layer was carried out simultaneously on both specimens by loading the samples together in the vacuum chamber. The estimation of the sputtering depth is discussed in the Supporting Information. The XAS spectra at the S L_2,3_-edge of CdS, CZTS and ACZTS were measured in electron yield mode using a pass energy of 75 eV. The energy scale was calibrated using a metal piece of Al foil. Prior to the measurements the samples were heated to 150 °C and/or a mild sputtering using an ion energy of 0.5–1 keV was performed. The surface cleaning was performed until clear signals from the surface were seen (such as vanishing of the O1s peak and onset of a clear Cd 4d peak in the case of CdS). The XPS peak fitting was carried out using XPST- X-ray Photoelectron Spectroscopy Tools, an IgorPro package for the analysis of the XPS data^19^.

## Results

### Theoretical background

XPS/UPS is a non-destructive tool to investigate the electronic properties of a semiconductor and can be used to determine heterojunction offsets, as demonstrated previously for CdS/CIGS^20^. To measure the energy band offset of a heterojunction, the valence band spectra can be monitored as a function of etching (or deposition) of a second semiconductor material^21^. The band offset and band bending are then monitored as a function of the overlayer thickness.When the VBO is large compared to the instrumental resolution, the valence band emission at the interface exhibits two valence-band edges which can be used to directly determine the VBO^9^.

With the alternatively and most commonly used approach, the VBO can be calculated by the difference in the binding energy of the cation core levels of the two semiconductors with respect to the valence-band maximum (VBM) position^14^. The VBO of n-CdS/p-CZTS heterojunction can be determined using the following equation:

1 $${\text{VBO}} = E_{{{\text{VBM}}}}^{{{\text{CZTS}}}} - E_{{{\text{VBM}}}}^{{{\text{CdS}}}} - E_{{{\text{ibb}}}} ,$$

where $E_{{{\text{VBM}}}}^{{{\text{CZTS}}}}$ and $E_{{{\text{VBM}}}}^{{{\text{CdS}}}}$ are the VBM of CZTS and CdS, respectively, and *E*_ibb_ is the interface-induced band bending. Given an interface position of the Fermi level, *E*_F_, the band-bending will extend on either side of the junction and on a length scale which depends on the doping level of the two semiconductors. Thus, *E*_ibb_ can be determined from the sum of the interface-induced changes due to band bending induced in CdS and CZTS^22,23^:

2 $$E_{{{\text{ibb}}}} = (E_{{{\text{Cd4d}}}}^{{{\text{CdS}}}} - E_{{{\text{Cd4d}}}}^{{\text{i}}} ) + \left( {E_{{{\text{Zn3d}}}}^{{\text{i}}} - E_{{{\text{Zn3d}}}}^{{{\text{CZTS}}}} } \right),$$

where $E_{{{\text{Cd4d}}}}^{{{\text{CdS}}}}$ and $E_{{{\text{Zn3d}}}}^{{{\text{CZTS}}}}$ are the binding energies of the Cd 4d and Zn 3d core levels of CdS and CZTS, respectively, while the index *i* denotes the interface.

Finally, the CBO can be estimated as:

3 $${\text{CBO}} = E_{g} \left( {{\text{CdS}}} \right) - E_{g} \left( {{\text{CZTS}}} \right) - {\text{VBO}},$$

where *E*_g_ (CdS) and *E*_g_ (CZTS) are the band gaps of CdS and CZTS, respectively.

### UPS depth profiles: an overview

**Figure 1:**
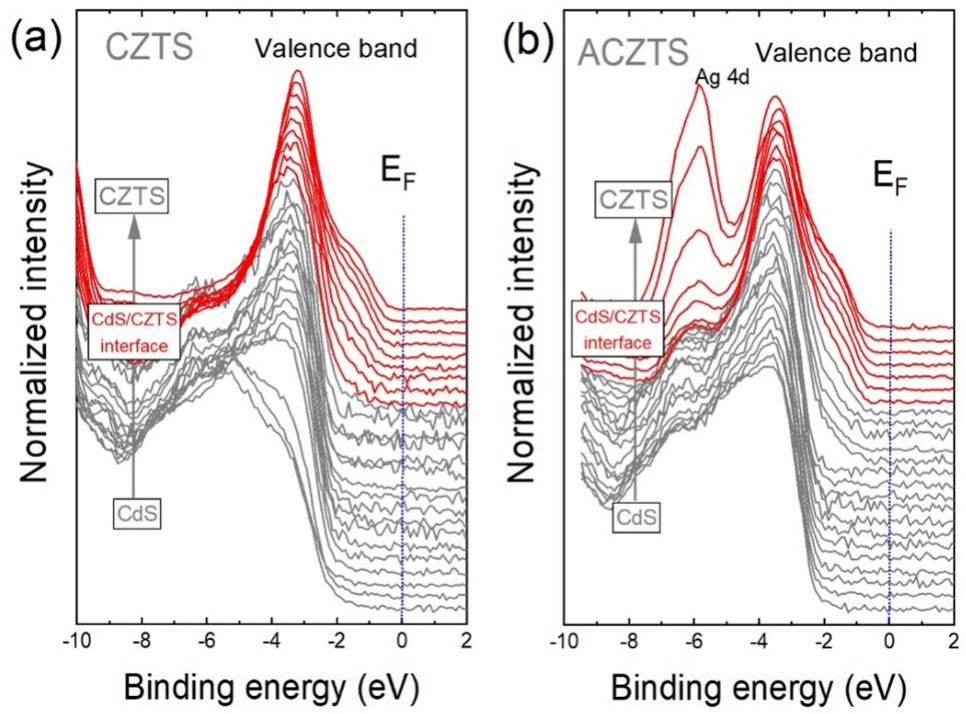
(**a**) UPS spectra at 60 eV taken at different sputtering depths across the CdS/CZTS (**a**) and CdS/ACZTS (**b**) heterostructures. The red plots indicate the interface region. E_F_ denotes the Fermi level.

**Figure 2:**
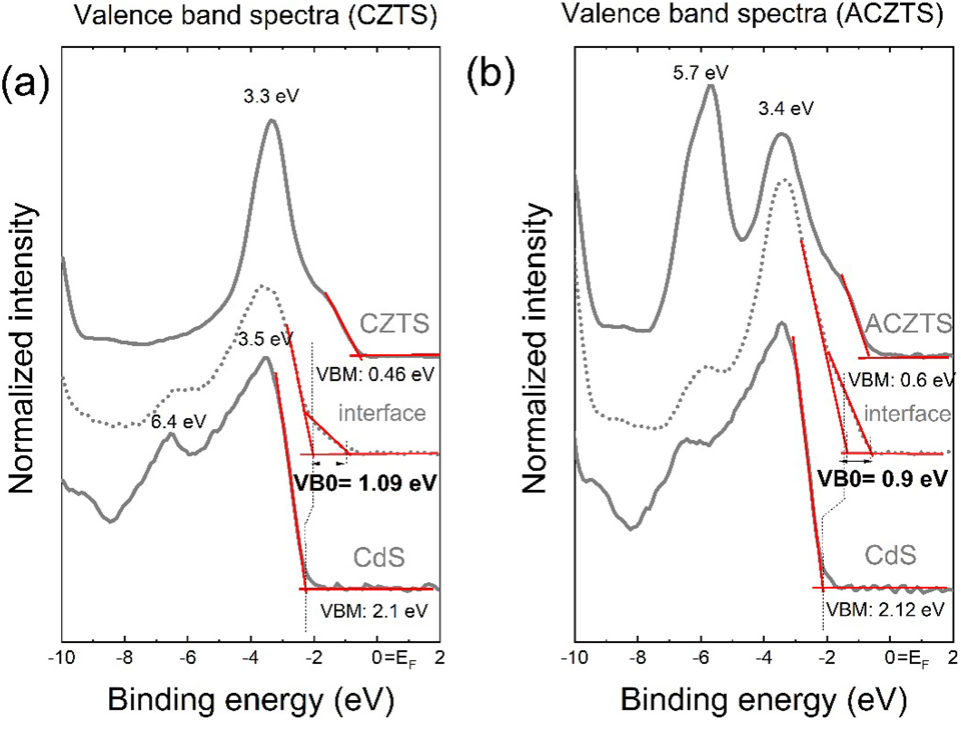
(**a**,**b**) UPS spectra of CdS, interface and CZTS/ACZTS. The term VBM denotes the valence band maximum position with respect with the Fermi level (E_F_), while the valence band offset (VBO) was determined from the difference in the VBM positions at the interface.

Figure 1 shows UPS spectra of CdS/CZTS and CdS/ACZTS as a function of the etching of the CdS layer. The sputtering parameters were converted to a global sputtering depth (arbitrary units), as described in the Supporting Information. The photoemission spectra were acquired using an excitation energy of 60 eV. Each spectrum represents the photoemission intensity (normalized units) in the energy range from 0 to 10 eV and reflects the density of states in the valence band. Here, the binding energy is referenced to *E*_F_ and all values below *E*_F_ are denoted as negative. The top of the valence band of the CdS layer consists primarily of a mixture of Cd 5 s and S 3p orbitals, and has two maxima at around 3.5 eV and 6.4 eV (see also Fig. 2), which is in good agreement with DFT calculations^24^. Upon gradual removal of the CdS layer, the photoemission spectra weight distribution changes and a broad shoulder appears in the energy range from 0.8 to 2 eV assigned to the valence band states of CZTS or ACZTS (red curves in Fig. 1). A striking difference between the UPS spectra of CZTS and ACZTS is the pronounced peak at a binding energy of ~ 5.7 eV, which appears upon Ag alloying of CZTS. This feature can be assigned to the emission from the Ag 4d core level. The top of the valence band of pure CZTS consists mainly of Cu 3d and S 3p hybrid orbitals, and upon partial substitution of Ag by Cu, Ag 4d orbitals hybridized with the S 3p orbitals are formed. Our experimental findings are in well agreement with DFT calculations, where a new band associated with the Ag 4d states located in the energy range from 4 to 6 eV is predicted to form.

It is worth mentioning that we do not observe a clear signature of the ion-beam induced damage of the CdS layer, which would inherently result in an emission spectra that extends near the E_F_ in the presence of metallic Cd^25^. In fact, ion beam sputtering has been undertaken as a common approach for the determination of the band alignment of ZnS/CIGS and CdS/CZTS heterojunctions^9,12,26,27.^

### Valence band offset: direct determination

If the valence band offset between the two semiconductors is large, which is the case of the CdS/CZTS heterostructure, the individual valence band edges can be resolved in the UPS spectra. In other words, the photoemission spectra at the interface can be regarded as a superposition of the substrate and overlayer-related valence band spectra.  Figure 2 shows the UPS spectra of three representative depths, i.e., bulk CdS (sputtering depth of 1000 a.u.), interface (sputtering depth of 2340 a.u.) and bulk CZTS/ACZTS (sputtering depth of 3500 a.u.). Since the photoelectron escape depth in CdS is 0.6 nm^28^, the photoemission spectra reflect entirely the band structure of the surface. The position of the VBM was determined by extrapolating the linear fit of the leading edge of the valence band to the background level^9^. The valence band edge of CdS ($E_{{{\text{VBM}}}}^{{{\text{CdS}}}}$) resides at − 2.1 ± 0.02 eV below the Fermi level, which is similar to the value reported for CdS in CdS/Cu_2_Zn(Sn_1−x_Ge_x_)Se_4_ (CdS/CZTGeSe)^29^. The valence band edge of CZTS ($E_{{{\text{VBM}}}}^{{{\text{CZTS}}}}$) was determined to be − 0.46 eV. The data indicate that the valence band edge of CZTS lowers by 0.14 eV upon Ag alloying ($E_{{{\text{VBM}}}}^{{{\text{ACZTS}}}} = - 0.6$ eV). This is in agreement with DFT calculations, which predict a down shift of the valence band edge of the Ag_2_ZnSnS_4_ (AZTS) by 0.74 eV with respect to that of CZTS^5^. This can be rationalized by the fact that the valence band of AZTS consists of Ag 4d states hybridized with S 3p states, and since the hybridized states of Ag 4d orbitals are localized deeper in the valence band (by 2 eV as compared to the Cu 3d states in pure CZTS), it would result in a lower valence band edge. In a crude approximation, and neglecting the band bending, VBOs of 1.64 eV and 1.52 eV were determined for CdS/CZTS and CdS/ACZTS, respectively.

Next, the valence band spectra at the interface are shown in Fig. 2, middle plots. As the top layer thickness decreases, the band bending at the interface will affect the local charge distribution resulting in a shift of the valence band edge of CdS. If we assign the high-energy band edge to the buffer layer and the low-energy edge to the absorber layer, we obtain VBO values of + 1.09 eV and + 0.9 eV for the CdS/CZTS and CdS/ACZTS heterostructures, respectively.

**Figure 3:**
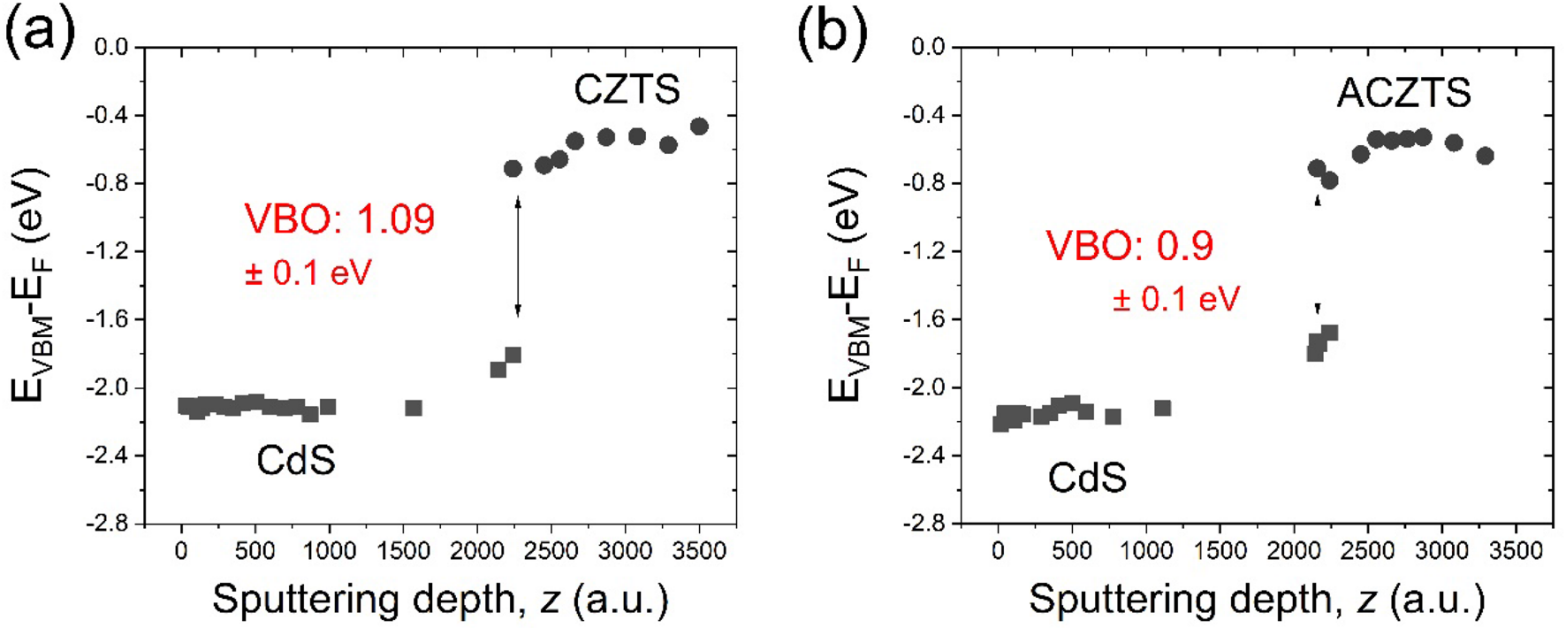
Valence band maximum with respect to the Fermi level (E_VBM_ − E_F_) as a function of sputtering depth, *z,* for the (**a**) CdS/CZTS and (**b**) CdS/ACZTS heterostructures. VBO was determined from the difference between the VBM of the respective semiconductors at the interface.

The evolution of the VBM as a function of the sputtering depth, *z*, for the CdS/CZTS and CdS/ACZTS heterostructures is shown in Fig. 3a,b, respectively. The data points were extracted from the experimental UPS depth profiles shown in Fig. 1. A substantial shift in the VBM of the CdS layer of ~ 0.3 eV to lower binding energy was determined from the difference between the VBM of 2.1 eV in the bulk to 1.8 eV at the interface. In case of the CdS/ACZTS system, the valence-band edge of CdS shifts by ~ 0.45 eV (from 2.12 eV in the bulk to 1.67 eV at the interface). For CZTS, the valence-band edge shifts by 0.25 eV to a higher binding energy from the interface to the bulk, while that of ACZTS shifts by 0.15 eV. At a depth above ~ 3500 a.u., the UPS spectrum is dominated by photoemission from CZTS or ACZTS. A VBO of 1.09 eV was determined for the CdS/CZTS and 0.9 eV for the CdS/ACZTS, from the difference in the VBM of the two semiconductors at the interface. Finally, the CBO value can be calculated using Eq. (3), assuming a band gap of 2.46 eV for bulk CdS, 1.49 eV for bulk CZTS and 1.57 eV for bulk ACZTS. The band gaps of CZTS and ACZTS were experimentally determined using the Kubelka–Munk formalism described previously^30^ (Figure S5, Supporting Information). This yields a CBO of − 0.12 eV and − 0.01 eV at the CdS/CZTS and CdS/ACZTS interfaces, respectively (see Table 1). We also note that, depending of the synthesis conditions, the surface band gaps of the buffer and absorber can differ from their bulk values^31^.

**Table 1:** Valence band offset (VBO) and conduction band offset (CBO) determined from the UPS spectra (direct method).

Sample	Valence band offset (VBO) at the interface(*) (eV)	Conduction band offset (CBO) (eV)
CdS/CZTS	+ 1.39	− 0.12
CdS/ACZTS	+ 1.26	− 0.01

All energy values are determined with respect to the Fermi level (E_F_).

*Values determined from UPS depth profiles at the interface.

### Interface-induced band bending

An alternative method for measuring band bending and thus indirectly determine the CBO is possible by means of XPS^20,32,33^, since the binding energy of the core levels at the interface shifts within the band bending zone as compared to the bulk, following a bending potential, *E*_ibb_. For a finite escape depth below 1 nm, the core level peaks can reflect the energy-shifted spectra of atoms at different levels across the band bending region, and thus a change in the surface potential across the depletion region. The indirect method is considered the most precise for high-accuracy measurements of heterojunction band discontinuities, Schottky-barrier heights, and interface band bending potentials^29^.

**Figure 4:**
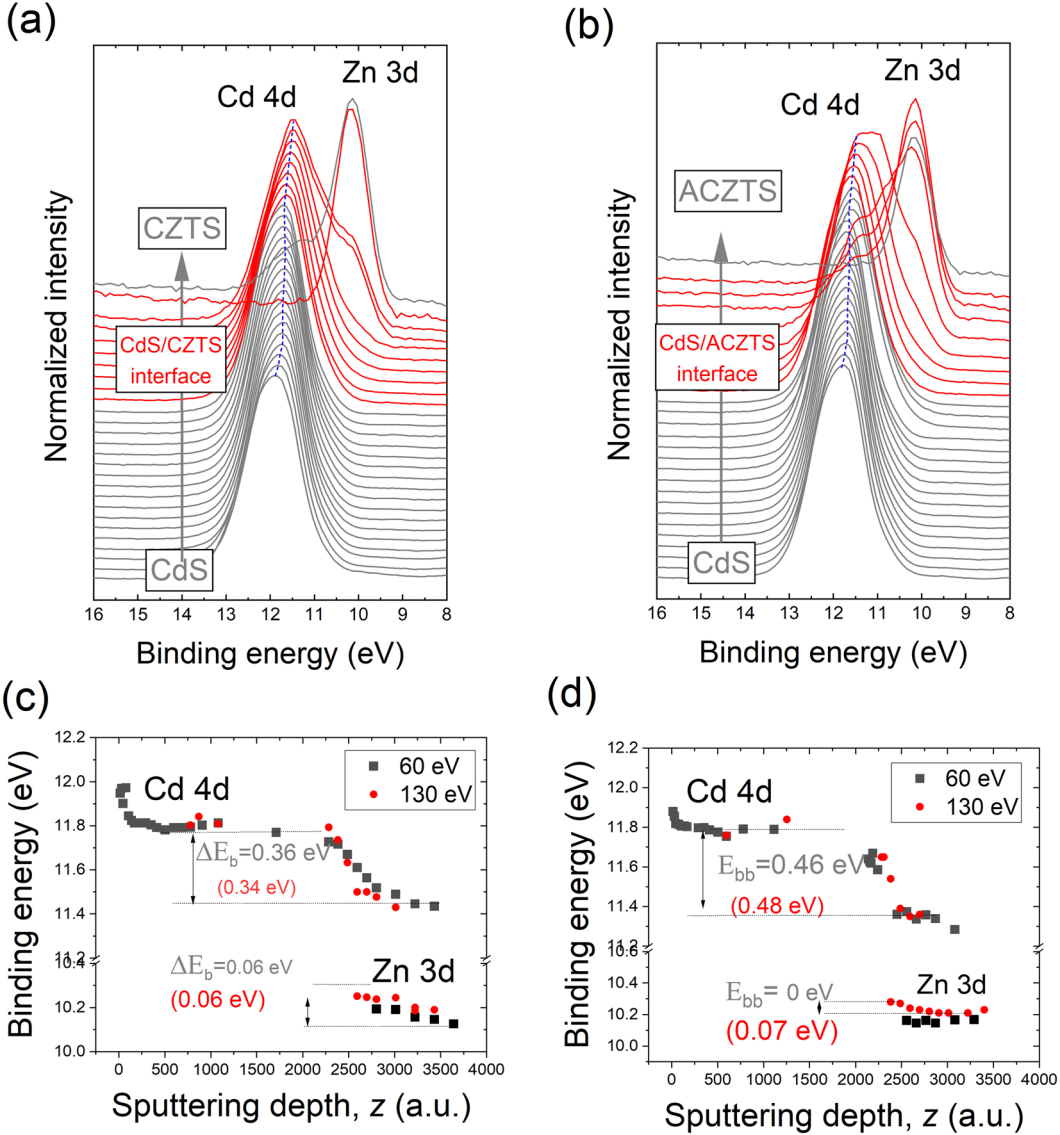
Depth-profile XPS spectra of CdS/CZTS (**a**) and CdS/ACZTS (**b**). The red-colored curves indicate the interface region; (**c**,**d**) The change in the binding energy (eV) of the Cd 4d and Zn 3d core levels as a function of sputtering depth for the CdS/CZTS (**c**) and CdS/ACZTS heterostructures (**d**). The binding energies were derived from XPS spectra taken at 60 and 130 eV.

The XPS/UPS spectra were measured at an energy of 60 eV, which corresponds to a large photoionization cross-section for both Cd 4d and Zn 3d subshells of 22.37 and 9.38 Mb, respectively^34^. Additionally, selected spectra were measured at an energy of 130 eV, which corresponds to a photoionization cross-section of 6.21 Mb of Zn 3d versus 0.2 Mb for Cd 4d^34^. The latter yields a higher signal for the Zn 3d peak as compared to the Cd 4d peak, and thus allows the identification of the CZTS or ACZTS layers with a higher sensitivity. The XPS spectra taken at 60 eV and at different depths across CdS/CZTS and CdS/ACZTS are shown in Fig. 4a,b, respectively. The S 2p XPS spectra are shown in Figure S2 of the Supporting Information. Since the spin–orbit coupling of the Cd 4d and Zn 3d peaks cannot be resolved, the spectra were fitted using only a single peak (Figure S1, Supporting Information). All spectra were fitted with Gaussian − Lorentzian functions after background removal according to the Tougaard method. The change in the binding energy of the Cd 4d and Zn 3d peaks as a function of the sputtering depth, *z,* across the CdS/CZTS and CdS/CZTS heterointerfaces is illustrated in Fig. 4c,d, respectively. We note a relatively good agreement between the XPS data points taken at 60 eV and 130 eV. Moreover, at an excitation energy of 130 eV, the Zn 3d peak can be distinguished closer to the interface (red dots in Fig. 4c,d). At a sputtering depth of ~ 3000 a.u., the Cd peak drops below the detection level and we interpret this as a complete removal of the CdS layer (CdS layer thickness equal to zero). The sputtering depth, *z* (a.u.), was then converted thickness, hence the top X-axis of Fig. 4c,d. The Cd 4d peak associated with the n-type CdS layer exhibits a substantial shift of 0.36 eV to a lower binding energy from the bulk to the interface region. The Zn 3d peak shifts by 0.06 eV from the interface to the p-CZTS layer. In the case of the CdS/ACZTS system, the Cd 4d peak shifts by 0.46 eV to a lower binding energy from the CdS to the interface, and the XPS reveals the presence of a thin layer at the interface. Since the binding energy of the peak varies between that of the Cd 4d peak and Zn 3d peak, the layer may be associated with a thin layer caused by intermixing at the CdS/ACZTS heterointerface (Figure S4, Supporting Information)**.**

The shift of the Cd 4d peak can be associated with i) interface band bending and/or ii) chemical shift due to intermixing between layers, presence of secondary phases, graded composition within the absorber. The full width of half-maximum (FWHM) of the peak estimated from the XPS spectra at 60 eV increases from 1.30 to 1.55 eV across the CdS/CZTS interface (Figure S3, Supporting Information). However, this apparent large peak broadening is partially due to the fact that the Cd and Zn peaks are not well resolved across the interface and the contribution of the Zn 3d peak to the peak tail of Cd 4d peak results in an overestimation of the peak width. In fact, the FWHM of the Cd 4d peak determined from XPS data taken at 130 eV (where the sensitivity of the Cd peak is much larger) was found to vary by ~ 0.1 eV across the interface. In the case of the CdS/ACZTS interface, the FWHM of the Cd 4d peak varies largely across the interface (by 0.2 eV, not shown here), and an intermixing layer is clearly resolved.

The substantial peak shift of the Cd 4d core level at the CdS/CZTS and CdS/ACZTS heterojunctions can be attributed to the interface-induced band bending, but a contribution from the chemical shift cannot be entirely excluded. The chemical shift may arrise from differences in stoichiometry of the n-CdS layer or/and chemical interaction at the interface, interdiffusion of Cd ions during the chemical-bath deposition process or etching.

**Figure 5:**
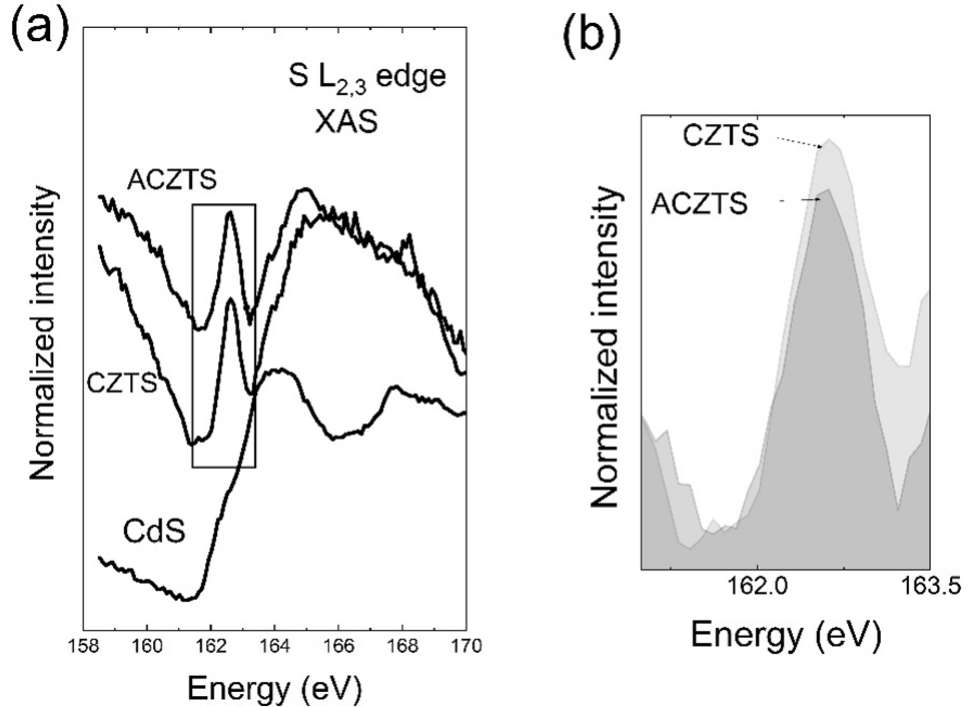
(**a**) The S L_2,3_ XAS spectra of CdS, CZTS and ACZTS obtained in electron yield mode. (**b**) Magnified image on given photon energy of the XAS spectra (normalized to the background level) indicating the decrease in the intensity of the peak at 163.4 eV upon Ag alloying.

Lastly, XAS allows probing the unoccupied states (conduction band states) in CZTS upon Ag alloying^35–37^. The XAS spectra were measured in electron yield mode (EY), as compared to the fluorescence yield (FY)-XAS which relies on the determination of the absorption coefficient from the intensity of the transmitted X-ray beam through a thin sample^14,36^. Due to the limited escape depth of the electrons, the EY-XAS spectra reflect exclusively the surface properties. Figure 5 shows the S L_2*,*3_ XAS spectra of the CdS, CZTS and ACZTS. The prominent peak at ~ 162.6 eV observed in the XAS spectra of CZTS and ACZTS represents bands derived from Sn- and/or S-related states associated with the bottom of the conduction band^35^. Moreover, we observe a decrease in the intensity of the peak at ~ 162.6 eV upon Ag alloying, as shown in the normalized XAS spectra in Fig. 5b. This can be associated with a weaker hybridization between the Sn 5 s and S 3p states upon Ag alloying. Indeed, since the newly formed Ag–S bonds in ACZTS are longer than the Cu–S bonds in pure CZTS (due the larger radius of Ag compared to Cu), this would result in a decrease in the hybridization between the Cu/Ag–S states.

## Discussion

**Figure 6:**
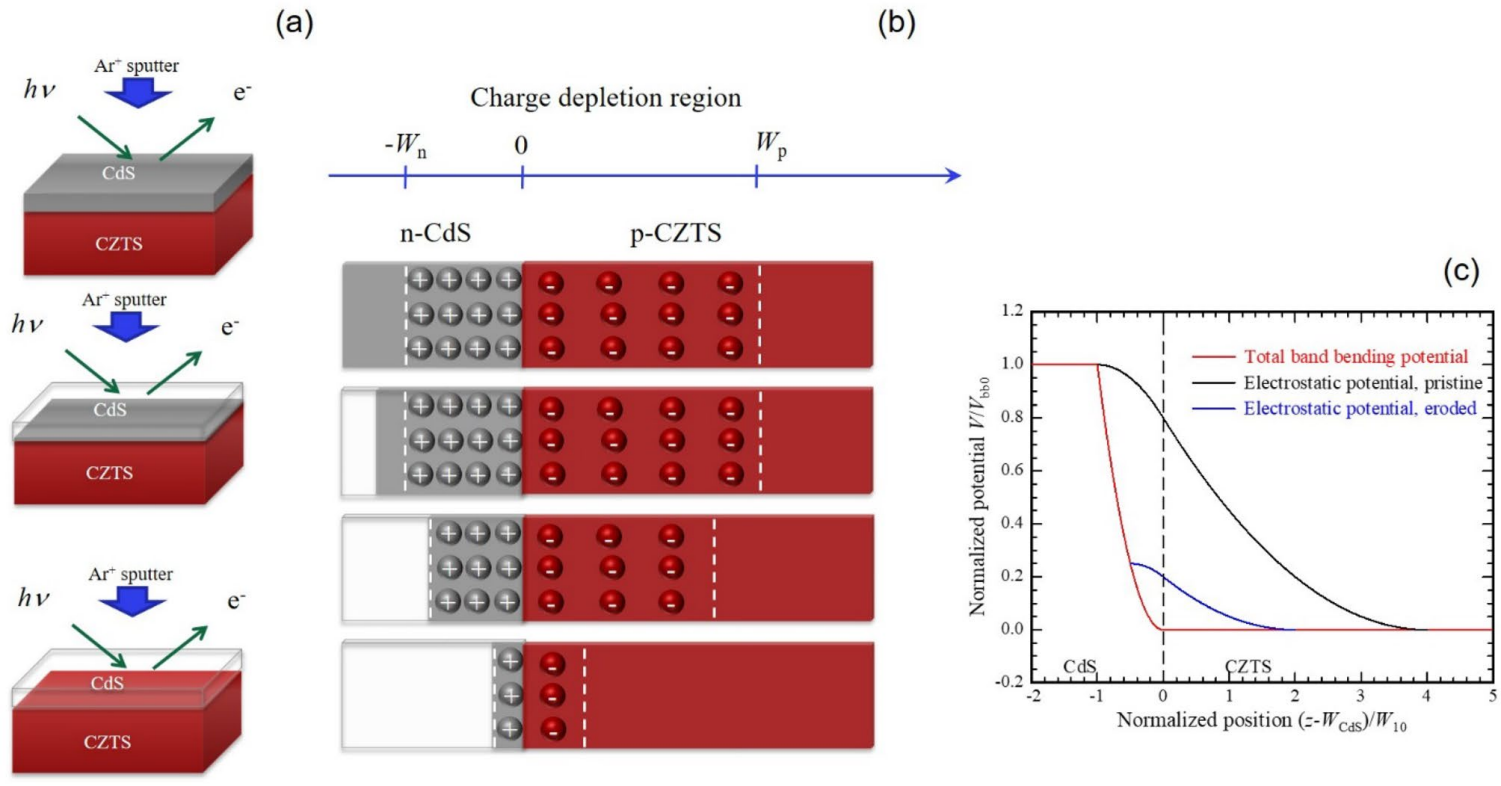
(**a**) Sketch of the ion beam sputtering of the CdS layer. (**b**) The evolution of the charge depletion layer at the CdS/CZTS interface as a function of etching of the top CdS layer. (**c**) Electrostatic potential, normalized to the total band bending potential *V*_bb0_, in the pristine CdS/CZTS device as a function of position, normalized by the CdS depletion width *W*_10_ (black curve). Normalized electrostatic potential of the eroded device calculated for a device with CdS eroded to a thickness of half the pristine CdS depletion layer thickness (blue curve). Normalized total band bending potential of the device during erosion as a function of normalized eroded surface position (red curve). This potential affects the UPS/XPS measurements due to the inherent surface sensitivity. The curves are calculated for a device that has a CZTS depletion width four times larger than the CdS depletion width, i.e., *W*_20_ = 4*W*_10_. *W*_CdS_ is the total CdS thickness of the pristine device, and the bottom axis is shifted by this amount in order to align the origin to the hetero-interface.

Our data reveal a significant shift of the Cd 4d peak associated with the n-CdS layer at the interface. Using the depletion approximation, one can consider the dependence of the electrostatic potential on the width of the charge depletion layer, as shown schematically in Fig. 6b,c. Awaw from the interface, the etching does not affect the charge neutrality region. However, when the width of the CdS layer becomes smaller than the pristine depletion layer thickness *W*_10_ on the n-CdS side, the depletion region begins to shrink. Consequently, the width of the depletion region on the p-CZTS side will shrink as well in order to maintain charge neutrality. The surface potential will vary with the width of the depletion layer and thus etching depth. The corresponding electrostatic potentials of pristine and eroded samples are shown in Fig. 6c. When *W*_CdS_ < *W*_10,_ such that the depletion region is exposed to vacuum, the surface potential (band bending voltages across both sides of the junction), *V*_bb_, will vary as $V_{{{\text{bb0}}}} \left( {\frac{{z - W_{{{\text{CdS0}}}} }}{{W_{10} }}} \right)^{2}$, where *V*_bb0_ is the initial surface potential, *W*_CdS0_ and *W*_10_ are the CdS thickness and depletion layer thickness of the pristine sample, respectively. Detailed calculations are given in the Supporting Information.

Here, we assume a shift in the binding energy of the Cd 4d core level of 0.42 eV and 0.53 eV for the CdS/CZTS and CdS/ACZTS heterojunctions, respectively due to a change in the surface potential across the depletion region (Table 2). Due to high surface sensitivity of the XPS at an energy 60 eV, the largest signal contribution arises from the top CdS layer, allowing for a nanometer-depth profiling of the surface potential at the interface. The width of the depletion region on the n-type CdS side, *W*_*n*,_ is roughly estimated to be around ~ 6.5 ± 2 nm. The widths of the depletion regions on the *n*- and *p*-type sides are related by: $\frac{{V_{{{\text{bbn}}}} \varepsilon_{n} }}{{W_{n} }} = \frac{{V_{{{\text{bbp}}}} \varepsilon_{p} }}{{W_{p} }}$ and $V_{{{\text{bbn}}}} \varepsilon_{n} N_{D} = V_{{{\text{bbp}}}} \varepsilon_{p} N_{A}$, where *V*_bb*n*_ and *V*_bb*p*_ are the band bending voltages on the either side of the heterojunction, *ε*_*n*_ and *ε*_*p*_ are the static dielectric constants of the n-CdS and p-CZTS semiconductors, respectively, *W*_*n*_ and *W*_*p*_ are the corresponding depletion widths, while *N*_*D*_ and *N*_*A*_ are the donor and acceptor densities. Since the doping density of the p-type CZTS is much larger than that of n-type CdS, band bending is expected to occur largely in the p-CZTS layer. Assuming *V*_bb*n*_ = 0.02 eV and $V_{{{\text{bb}}p}} = 0.40\,{\text{eV}}$, such as the total the surface potential is 0.42 eV, and *ε*_*n*_ = 10.2 and *ε*_*p*_ = 7, we estimate a width of the depletion layer on the p-CZTS side of ~ 89 ± 20 nm. The estimated total depletion width of CZTS ~ 95.5 ± 20 nm is in good agreement with previous data^38,39^. In the case of ACZTS, assuming *V*_bb*n*_ = 0.51 eV and *V*_bb*p*_ = 0.02 eV, a total of the depletion width of *W*_ACZTS_ ~  ~114 (± 20) nm is obtained. The data suggests that the total charge-depletion width in CdS/ACZTS is significantly wider than in CdS/CZTS. Furthermore, the doping concentration of the p-type layer $N_{A} = 2\frac{{\varepsilon_{0} }}{e}V_{bb} /W_{p} \left( {\frac{{W_{n} }}{{\varepsilon_{n} }} + \frac{{W_{p} }}{{\varepsilon_{p} }}} \right)$ was estimated to be ~ 1.2 × 10^16^ cm^−3^ for CZTS and ~ 9.73 × 10^15^ cm^−3^ for ACZTS. For CdS, a doping concentration of ~ 1.67 × 10^17^ cm^−3^ was determined.

**Table 2:** The interface band bending values, E_ibb_, valence band maximum of CdS ($E_{{{\text{VBM}}}}^{{{\text{CdS}}}}$), CZTS ($E_{{{\text{VBM}}}}^{{{\text{CZTS}}}}$), ACZTS ($E_{{{\text{VBM}}}}^{{{\text{ACZTS}}}}$), VBO and CBO values determined from the depth profile XPS spectra of the CdS/CZTS and CdS/ACZTS layers (indirect method).

Sample	*E*_ibb_ (ev)	$E_{{{\text{VBM}}}}^{{{\text{CZTS}}}} /E_{{{\text{VBM}}}}^{{{\text{ACZTS}}}}$(ev)	$E_{{{\text{VBM}}}}^{{{\text{CdS}}}}$(ev)	Valence band offset (VBO) (ev)	Conduction band offset (CBO) (ev)
CdS/CZTS	+ 0.42	− 0.46	− 2.10	+ 1.22	− 0.25
CdS/ACZTS	+ 0.53	− 0.6	− 2.12	+ 1.00	− 0.11

All energy values are determined with respect to the Fermi level.

**Figure 7:**
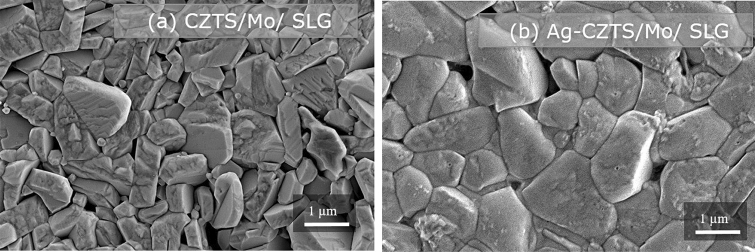
Top-view SEM images of (**a**) CZTS and (**b**) Ag-CZTS films with metal ratio Ag/(Ag + Cu) ~ 0.5 grown by PLD.

This study indicates that the incorporation of Ag in the kesterite lattice lowers the valence-band edge of CZTS with respect to the Fermi level. More importantly, the nano-scale depth profiling of the surface potential reveals a larger band bending at the interface and a significantly larger total charge depletion width at the CdS/ACZTS heterojunction as compared to CdS/CZTS. The latter suggests that ACZTS solar cells can benefit from a larger depletion width which can increase the probability of charge carrier collection in a solar cell. Moreover, Ag alloying of CZTS enhances significantly the grain size, as revealed by the secondary electron microscopy (SEM) images in Fig. 7.

**Figure 8:**
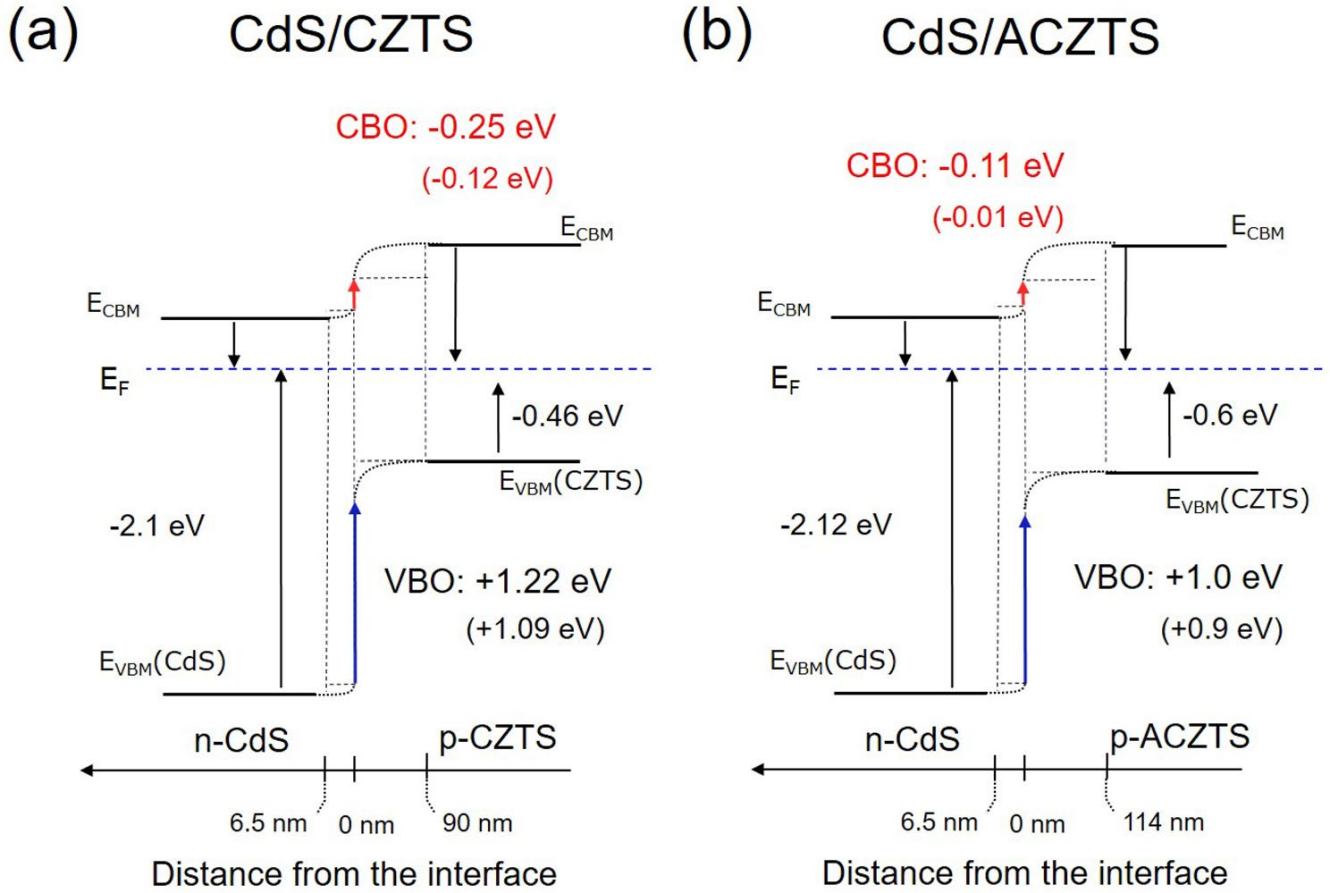
Band alignment at the (**a**) CdS/CZTS and (**b**) CdS/ACZTS heterointerfaces.

The schematic diagrams of the band alignment at the heterointerface between the n-CdS/p-CZTS and n-CdS/p-ACZTS are illustrated in Fig. 8a,b, respectively. The VBO determined in this work of 1.22 eV for CdS/CZTS is comparable with previous reports of 1.20 eV^15^ and 1.24 (± 0.14) eV^27^ and with the theoretical predicted values of 1.24 eV^40^. When the direct method is used, the determined VBO values are slightly lower (+ 1.09 eV for CdS/CZTS). Small offsets between VBO values estimated from the direct versus the indirect method were consistently reported in the literature^29^. Neverthenless, both direct and indirect methods show the same trend, i.e., VBO at the CdS/CZTS interface is reduced after Ag alloying.

Finally, CBO values of − 0.25 eV and − 0.11 eV were determined for CdS/CZTS and CdS/ACZTS, respectively. The negative conduction offsets reveal a cliff-like band alignment at both heterointerfaces. According to device simulations, the cliff can act as a barrier against injected electrons at forward bias, resulting in recombination of majority carriers via defects at the interface^14,15,27,41^. Neverthenless, with decreasing the absolute value of the conduction band offset in the case of CdS/ACZTS, the barrier is lowered, and this can ultimately result in an increase in Voc and FF in ACZTS devices.

## Conclusions

We have shown that depth-profile XPS and UPS analysis of the CdS/CZTS and CdS/Ag-CZTS heterostructures can reveal detailed information on the nanometer-scale evolution of the valence band structure at the interface. The valence band maximum position of CdS undergoes a substantial shift across the heteinterfaces, which can be attributed to band bending at the interfaces and chemical shift. Ag alloying of CZTS lowers the valence-band maximum of CZTS with respect with Fermi level. Using a model based on charge neutrality conservation, we have estimated a total depletion width of ~ 95.5 ± 20 nm for CdS/CZTS and ~ 114 (± 20) nm for CdS/ACZTS. Conduction band offsets of − 0.25 eV for CdS/CZTS and − 0.11 eV for CdS/ACZTS were determined. This methodology allows for investigating the nano-scale electronic properties of the CdS-kesterite junctions, and can bring a step forward on the development of kesterite solar cells utilizing either alloyed kesterites or alternative buffer layers. It can be applied as an universal approach to unveil the interfacial electronic properties of any type of heterostructures which exhibit synergetic properties owing to the formation of the heterointerfaces.

## Supporting information

Details on the Ar^+^ ion sputtering of the CdS layer; Detailed calculations on the evolution of the charge depletion layer at the CdS/CZTS and CdS/Ag-CZTS interface as a function of etching of the CdS layer; XPS peaks fittings of the Cd 4d and Zn 3d peaks measured at different depths across the CdS/CZTS and CdS/ACZTS interfaces; XPS fittings of the S 2p peaks across the CdS/CZTS and CdS/ACZTS interfaces; FWHM and binding energy values of the Cd 4d peaks across the CdS/CZTS and CdS/ACZTS layers; Determination of the optical band gap of CZTS and Ag-CZTS using Kubelka–Munk formalism.

## Supplementary information

Supplementary information.
