# Supplementary material for: Energy band alignment at the heterointerface between CdS and Ag-alloyed CZTS
Source: Sci Rep. 2020 Oct 27;10:18388. doi: 10.1038/s41598-020-73828-0 (PMC7591932; doi:10.1038/s41598-020-73828-0)
Supplement: Supplementary file 1 — Supplementary information. [file 41598_2020_73828_MOESM1_ESM.docx]

Supplementary Information

**Energy band alignment at the heterointerface between CdS and Ag-alloyed CZTS**

Mungunshagai Gansukh^†^, Zheshen Li^‡^, Moises Espindola Rodriguez^||^, Sara Engberg^†^, Filipe Mesquita Alves Martinho^†^, Simon López **Mariño**^§^, Eugen Stamate^§^, Jørgen Schou^†^, Ole Hansen^§^ and Stela Canulescu†^*^

*†Department of Photonics Engineering, Technical University of Denmark, DK-4000 Roskilde, Denmark*

*‡ ISA, Department of Physics and Astronomy, Aarhus University, DK-8000 Aarhus C, Denmark*

*^||^Department of Energy Conversion and Storage, Technical University of Denmark, DK-4000 Roskilde, Denmark*

*§DTU Nanolab, Technical University of Denmark, DK-2800 Kgs. Lyngby, Denmark*

*Corresponding author: Stela Canulescu, email [*stec@fotonik.dtu.dk*](mailto:stec@fotonik.dtu.dk)

Supplementary Note 1. Sputtering depth.

The sputtering depth, defined the number of sputtered atoms per time unit, can be estimated as:

$R=\frac{YJ}{q_{e}}$, (1)

where$Y$ is the sputtering yield (number of sputtered particles per incoming ion),$J$is the beam current on target and $q_{e}$the elementary charge^1^. $Y$ depends on the energy of the Ar^+^ ions, the angle of incidence and on the volatility (the cohesive energy) of the sputtered material. Since $J$ is known from instantaneous measurements, and the angle of incidence is fixed, the sputtering rate depends in the present case only on the ion energy and on the cohesive energy of the individual atoms in the sulfides. For an ion energy ranging between 0.5 and 1 keV (see Table 1), the yield can be considered a linear function of the energy, such that the depth can be determined in relative units.

| Ion energy | Angle of incidence | Ion current |
| --- | --- | --- |
| 0.5-1 keV | 40 degrees | 5.65-10 μA |

**Table S1**. The sputtering parameters used for the XPS/UPS depth profiling

Supplementary Note 2. **Depth profiling of the p-n heterojunction**

The magnitude of the charge present on either sides of the p-n junction is given by:

$Q=eN_{D}W_{n}$, (2)

$Q{=eN}_{A}W_{p}$, (3)

where $N_{D}$ and $N_{A}$ are the doping density on the n-CdS and p-CZTS sides, respectively and *e* is the elementary charge.

The corresponding maximum electric field magnitudes are:

$E_{n\max}=\frac{eN_{D}W_{n}}{\varepsilon_{0}\varepsilon_{n}}$, (4)

and

$E_{\boldsymbol{p}\max}=\frac{eN_{A}W_{p}}{\varepsilon_{0}\varepsilon_{p}}$, (5)

where $W_{n}$ and $W_{p}$ are the charge depletion layer widths on the n-CdS and p-CZTS sides, respectively, $\varepsilon_{0}$is the permittivity of vacuum and $\varepsilon_{n}$ and $\varepsilon_{p}$ are the static dielectric constants of the n- and p-type semiconductors.

The total band bending voltage across the junction is:

$V_{\mathrm{bb}}$=$V_{\mathrm{bb}n}+V_{\mathrm{bb}p},$ (6)

or

$V_{\mathrm{bb}}=\frac{1}{2}E_{n\max}W_{n}+\frac{1}{2}E_{p\max}W_{p}$ (7)

Combining eqs. (4-6), the total band bending voltage becomes:

$V_{\mathrm{bb}}$**=**$\frac{\boldsymbol{1}}{\boldsymbol{2}}\frac{eN_{D}}{\varepsilon_{0}\varepsilon_{n}}W_{n}^{2}$(1+$\frac{\varepsilon_{n}}{\varepsilon_{p}}\frac{N_{D}}{N_{A}}),$ (8)

or

$V_{\mathrm{bb}}=\frac{\boldsymbol{1}}{\boldsymbol{2}}\frac{eN_{D}}{\varepsilon_{0}\varepsilon_{n}}W_{n}^{2}$(1+$\frac{\varepsilon_{n}}{\varepsilon_{p}}\frac{W_{p}}{W_{n}})$ (9)

where the eq. (8) denotes the band bending voltage across the n-type top-layer while eq. (9) is that across the p-type absorber, i.e.,

$V_{\mathrm{bb}n}$**=**$\frac{\boldsymbol{1}}{\boldsymbol{2}}\frac{eN_{D}}{\varepsilon_{0}\varepsilon_{n}}W_{n}^{2}$, (10)

and

$V_{\mathrm{bb}n}$**=**$\frac{\boldsymbol{1}}{\boldsymbol{2}}\frac{eN_{D}}{\varepsilon_{0}\varepsilon_{n}}W_{n}^{2}\frac{\varepsilon_{n}}{\varepsilon_{p}}\frac{W_{p}}{W_{n}}$ (11)

Thus, the band bending voltages across the p- and n-sides of the junction are related as:

$\frac{V_{\mathrm{bb}n}\varepsilon_{n}}{W_{n}}=\frac{V_{\mathrm{bb}p}\varepsilon_{p}}{W_{p}}$ (12)

or

$V_{\mathrm{bb}n}\varepsilon_{n}N_{D}=V_{\mathrm{bb}p}\varepsilon_{p}N_{A}$ (13)

In the case of the CdS/CZTS hetero-structure, where$N_{D}>N_{A}$, the potential drop across the CZTS layer is larger than that across the CdS layer.

When the top n-CdS layer is etched away, the surface potential will vary depth, as discussed below.

When the sputtering depth,$z$ <$W_{\mathrm{top}}-W_{10}$, such that a charge neutral top layer still exists, the pristine band bending voltage (the pristine surface potential) is:

$V_{\mathrm{bb}0}$**=**$\frac{\boldsymbol{1}}{\boldsymbol{2}}\frac{eN_{D}}{\varepsilon_{0}\varepsilon_{n}}W_{10}^{2}$(1+$\frac{\varepsilon_{n}}{\varepsilon_{p}}\frac{N_{D}}{N_{A}})$, (14)

where $W_{10}$ is the pristine depletion layer thickness and $W_{\mathrm{top}}$ is the pristine top-layer thickness.

When z > $W_{\mathrm{top}}-W_{10}$, such that the charge-depletion region is exposed to vacuum, the depletion layer shrinks during erosion, which results in the surface potential (or band bending voltage):

$V_{\mathrm{bb}}$**=**$\frac{\boldsymbol{1}}{\boldsymbol{2}}\frac{eN_{D}}{\varepsilon_{0}\varepsilon_{n}}({W_{\mathrm{top}}-z)}^{2}$(1+$\frac{\varepsilon_{1}}{\varepsilon_{2}}\frac{N_{D1}}{N_{A2}})$ (15)

${V_{\mathrm{bb}}=V}_{bb0}\frac{{(W_{\mathrm{top}}-z)}^{2}}{W_{10}^{2}}$ (16)

Eq. (16) indicates that the surface potential will drop to zero when *z*=$W_{\mathrm{top}}$, as shown schematically in Fig. 6.

Supplementary Note 3. **XPS curve fitting**


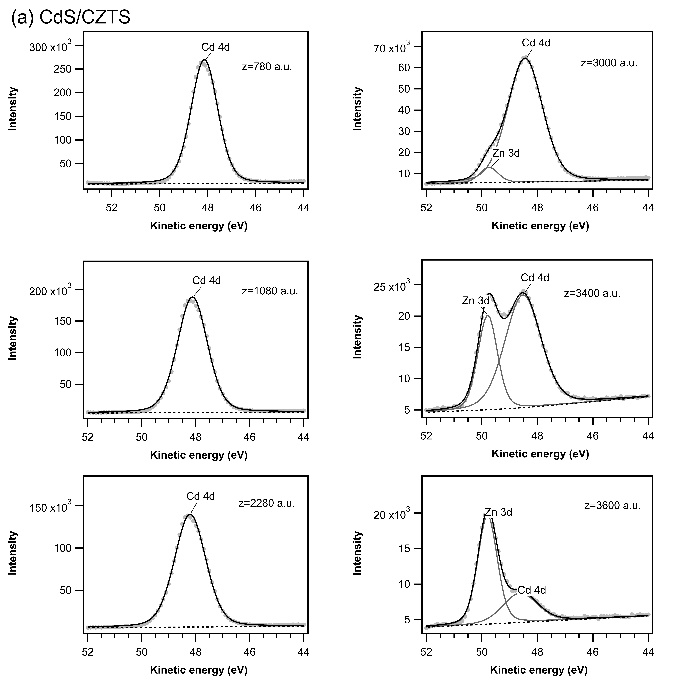

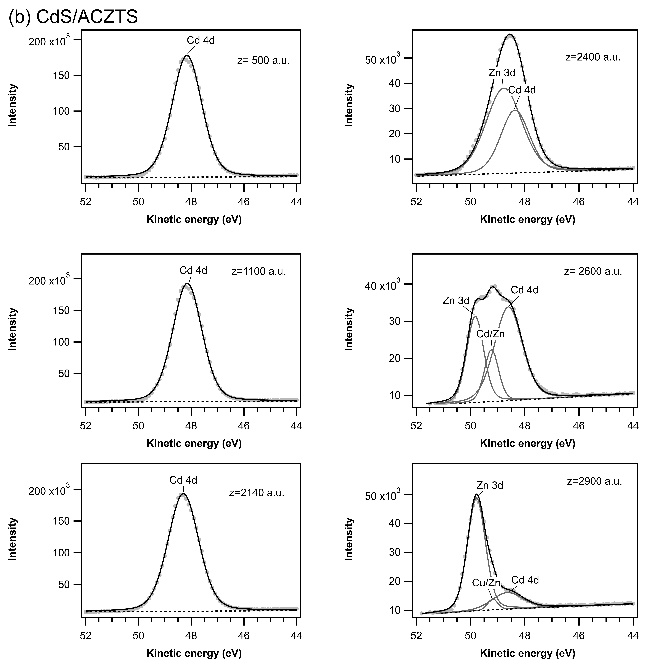


**Figure S1.** Cd 4d and Zn 3d XPS core level peaks measured at different sputtering depths across **(a)** CdS/CZTS and **(b)** CdS/ACZTS. Here *z* (a.u.) denotes the etching depth. All spectra were recorded using an energy hν=60 eV. Peak fitting was performed with the XPST tool available in the Igor Pro software.

Figure S1 shows the Cd 4d and Zn 3d peaks corresponding to the CdS and absorber layers, respectively. In the case of the CdS/ACZTS system, selected spectra at the interface were fitted with three peaks and the intermediate peak can be associated with a very thin mixed layer at the interface.


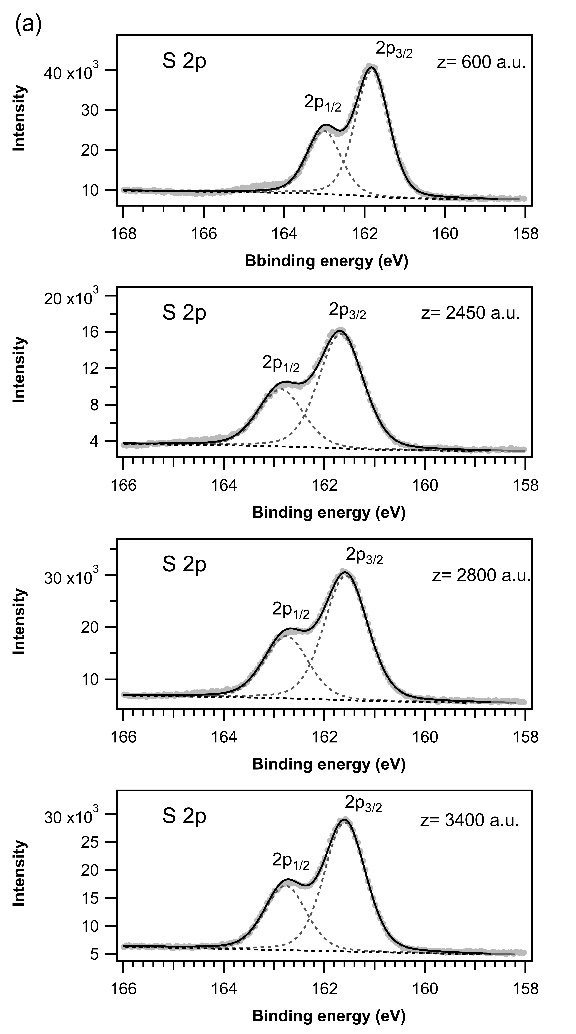

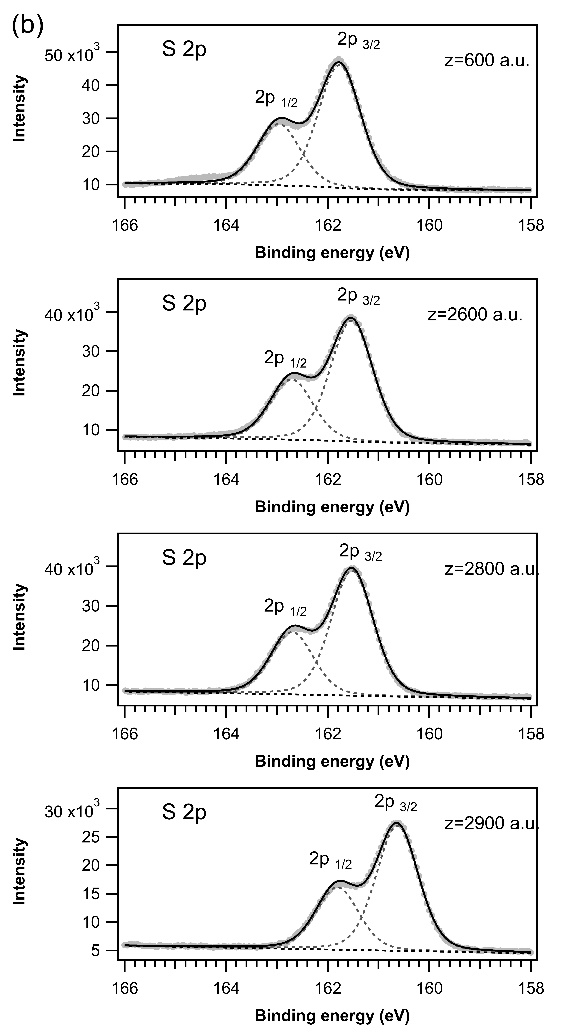


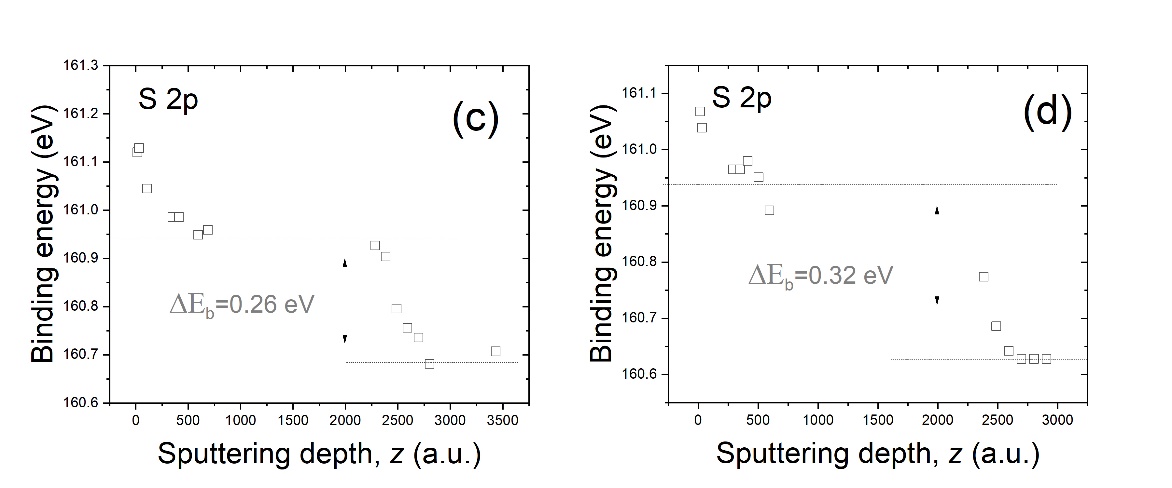


**Figure S2.** S 2p XPS peaks (including peak fittings) at different etching depths across the **(a)** CdS/CZTS and **(b)** CdS/AZCTS interfaces. The binding energy of the S 2p peak as a function of the sputtering depth for **(c)** Cd/CZTS and **(d)** CdS/ACZTS.


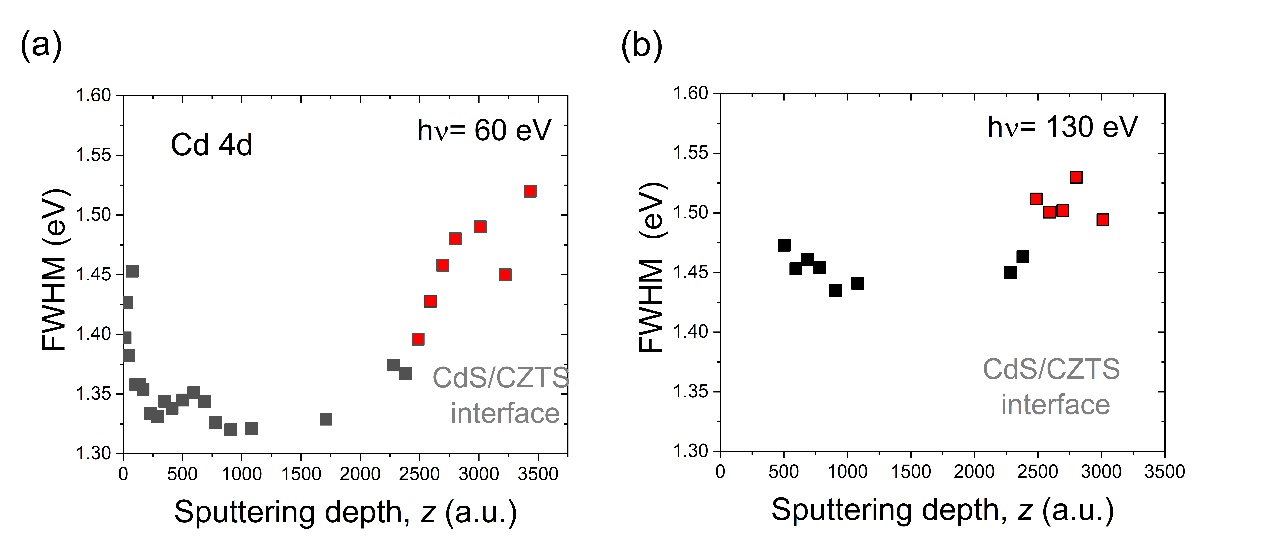


**Figure S3** The evolution of the full width of half-maximum (FWHM) of the Cd 4d peak as a function of the sputtering depth. The gray points denote the CdS bulk while the red points the CdS/CZTS interface. XPS data taken at an excitation energy of (a) 60 eV and (b) 130 eV.


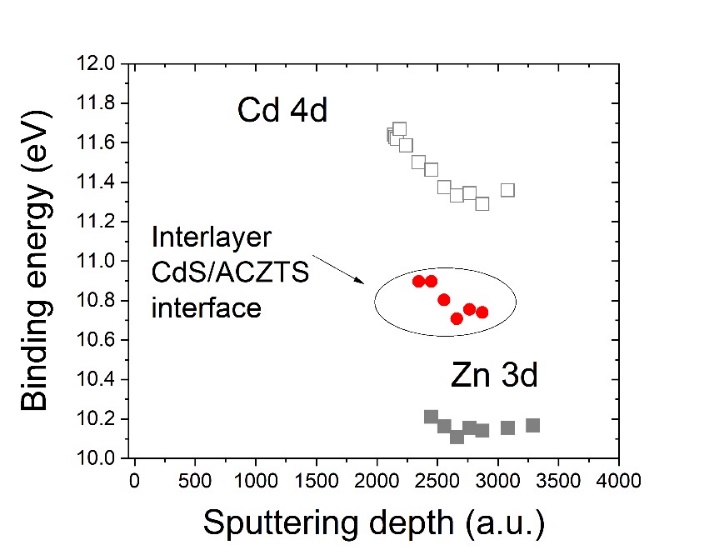


**Figure S4.** Binding energy of the Cd 4d, mixed layer and Zn 3d XPS core levels across the CdS/ACZTS heterointerface.

Supplementary Note 4. **Determination of the optical band gap using Kubelka-Munk formalism**


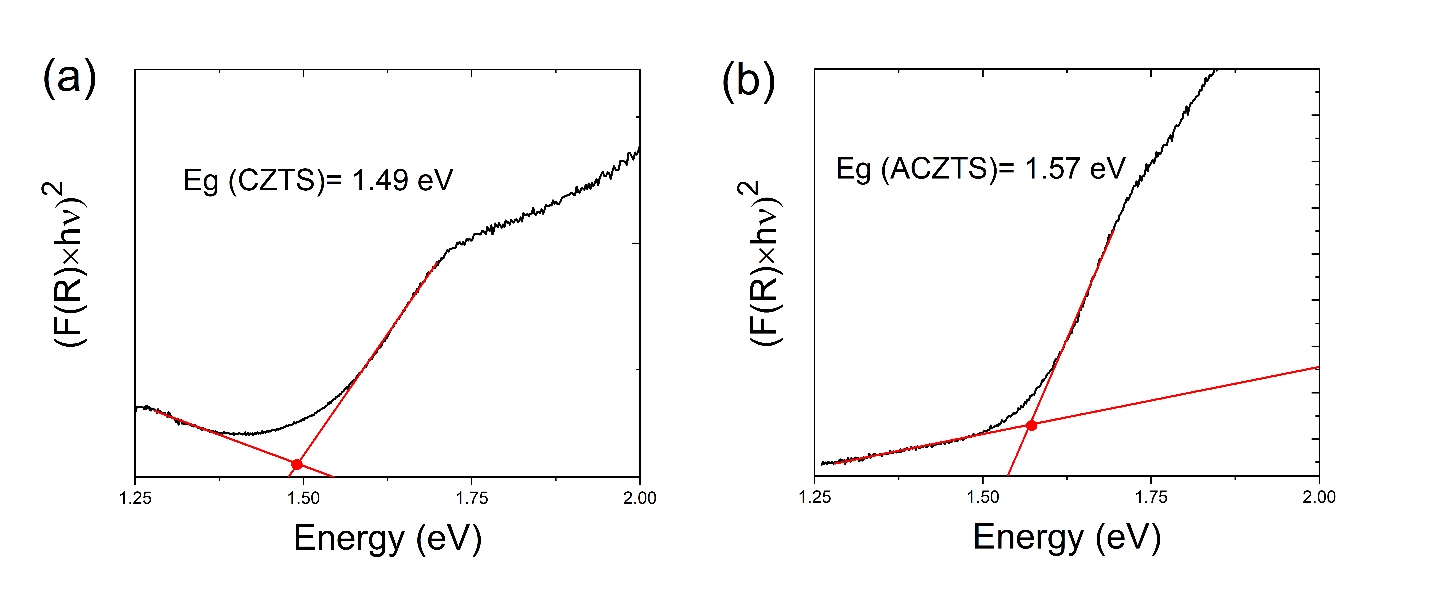


**Figure S5.** Optical band gap of **(a)** CZTS and **(b)** ACZTS absorbers determined from the Kubelka-Munk formalism^2^.

The optical band gap of the CZTS and ACZTS films on Mo/SLG substrates was determined using the Kubelka-Munk formalism, as described previously^2^, and assuming direct band to band optical transitions. The optical band gaps of 1.49 ± 0.01 eV and 1.57 ± 0.01 eV were determined for CZTS and ACZTS, respectively.

**REFERENCES**

1. Smith, D. L. *Thin-Film Deposition: Principles and Practice*. (MGraw-Hill, 1995).

2. Davídsdóttir, S. *et al.* Interfacial structure and photocatalytic activity of magnetron sputtered TiO2 on conducting metal substrates. *ACS Appl Mater Interfaces* **6**, 22224–22234 (2014).
